# Primary diffuse large B cell lymphoma arising from a leiomyoma of the uterine corpus

**DOI:** 10.1186/s13000-016-0464-8

**Published:** 2016-01-20

**Authors:** Lianhua Zhao, Qiang Ma, Qiushi Wang, Ying Zeng, Qingya Luo, Hualiang Xiao

**Affiliations:** Department of Pathology, Daping Hospital and Research Institute of Surgery, Third Military Medical University, Chongqing, China

**Keywords:** Diffuse large B cell lymphoma, Leiomyoma, Collision tumor

## Abstract

**Background:**

Primary diffuse large B cell lymphoma (DLBCL) of the uterus is rare, and primary DLBCL arising from a uterine leiomyoma (collision tumor) has not been reported in the literature.

**Case presentation:**

We describe the clinical, histological, immunohistochemical, and molecular features of primary DLBCL arising from a leiomyoma in the uterine corpus. A 73-year-old female patient had a uterine mass for 23 years. An ultrasound scan revealed marked enlargement of the uterus, measuring 18.2 × 13 × 16.3 cm, with a 17.6 × 10.9 × 11.6 cm hypoechoic mass in the uterine corpus. The tumors consisted of medium- to large-sized cells exhibiting a diffuse pattern of growth with a well-circumscribed leiomyoma. The neoplastic cells strongly expressed CD79α, CD20 and PAX5. Molecular analyses indicated clonal B-cell receptor gene rearrangement.

**Conclusions:**

To the best of our knowledge, no previous cases of primary DLBCL arising from a leiomyoma have been reported. It is necessary to differentiate a diagnosis of primary DLBCL arising from a leiomyoma from that of leiomyoma with florid reactive lymphocytic infiltration (lymphoma-like lesion). Careful analysis of clinical, histological, immunophenotypic, and genetic features is required to establish the correct diagnosis.

## Background

Primary lymphoma of the uterus is rare and diffuse large B cell lymphoma (DLBCL) is the most common histological subtype [1, 2]. However, primary DLBCL arising from the uterine leiomyoma has not been reported in the literature. We describe, for the first time, a collision tumor consisting of DLBCL and leiomyoma of the uterus in a 73-year-old female patient. To the best of our knowledge, no previous report has described the concurrence of these two types of tumors in any other tissues or organs.

## Case presentation

A 73-year-old woman had a uterine mass for 23 years without abnormal vaginal bleeding or discharge. A sonographic examination performed 23 years prior showed a myomatous mass that measured approximately 7 cm (as described by the patient; no ultrasound report was available). The patient did not complain of discomfort and did not undergo a Pap smear or medical treatment during this 23-year period. One year ago, the patient underwent a gynecological B ultrasound examination, which revealed that the uterine mass had enlarged since the previous sonogram (as described by the patient; no ultrasound report was available). The more recent ultrasound scan revealed marked enlargement of the uterus, measuring 18.2 × 13 × 16.3 cm, with a 17.6 × 10.9 × 11.6 cm hypoechoic mass in uterine corpus. This mass was nonhomogeneous and had irregular borders. The cervical thickness was 2 cm and had homogeneous echogenicity. The endometrial thickness was 3 mm. Bilateral adnexa were not clearly delineated. The mass was diagnosed as a leiomyoma by ultrasound. Laboratory tests revealed that the serum CA-153 level was 39.93 U/ml (high), the β2-microglobulin level was 3.24 mg/L (high), and the serum lactate dehydrogenase level and other biochemical test results were normal. The complete blood cell count revealed a white blood cell count of 6.4×10^9^ /L, a hemoglobin level of 123 g/L, and a platelet count of 186×10^9^/L. The Pap smear results showed moderate inflammation and no other abnormalities. Intraoperatively, a well-defined intramural myoma was observed in the anterior wall of the uterine fundus (The surgeon opened the uterus but did not collect a sample during the surgery for generation of frozen sections), and hysterectomy and bilateral adnexectomy were performed. The postoperative course of the patient was uneventful. The patient underwent no further examination or treatment due to economic reasons. At the most recent follow-up (13 months after the diagnosis), the patient was alive and did not report any discomfort.

**Fig. 1:**
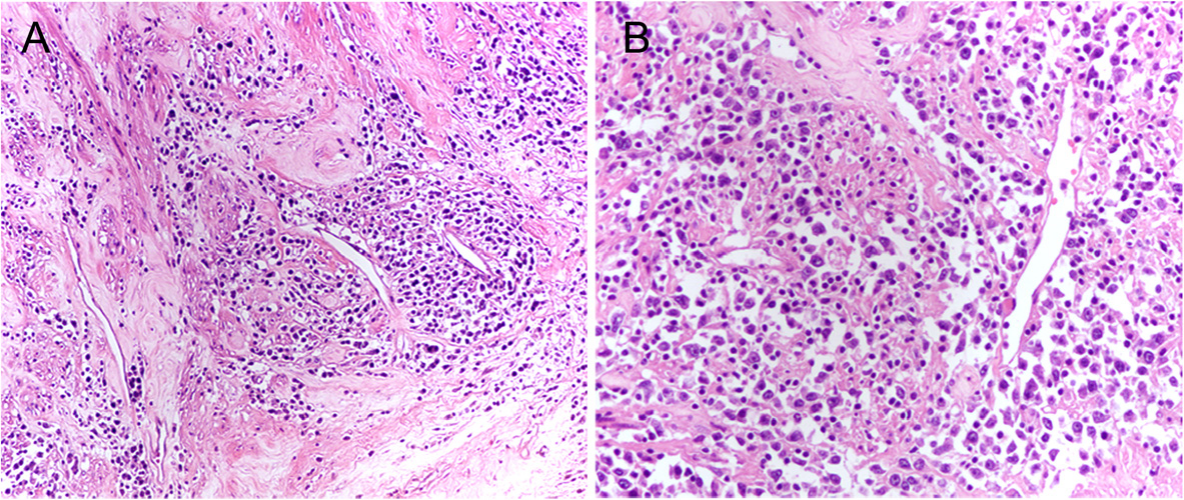
Histological features of primary DLBCL arising from a leiomyoma of the uterine corpus. Diffuse proliferation of lymphoid cells dissecting the muscle fiber is observed (**a**). Lymphoid cells consist of medium- to large-sized cells with abundant, faintly basophilic cytoplasm and round to oval nuclei, which are occasionally convoluted and which contain dispersed nuclear chromatin and one to multiple prominent nucleoli (**b**). [A-B: hematoxylin and eosin (H & E) staining; A: original magnification (O.M.), 100×; B: O.M., 200×]

**Fig. 2:**
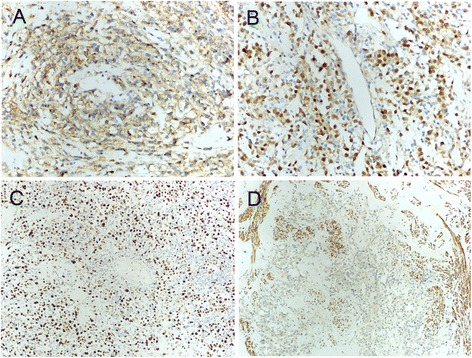
Immunohistochemical findings of primary DLBCL arising from a leiomyoma of the uterine corpus. Most lymphoid cells are positive for CD20 (**a**) and PAX5 (**b**). The proliferative index is high (approximately 50 %) (**c**). Smooth-muscle cells are positive for caldesmon (**d**). (A: CD20 stain, B: PAX5 stain, C: Ki-67 stain, D: caldesmon stain; A, B: O.M., 200×; C, D: O.M., 100×)

On gross examination, the uterine corpus was completely replaced by a whorled intramural single mass with a diameter of 17 cm, and the cut surface appeared yellowish with focal hemorrhage, without any necrosis or calcification. The endometrium was smooth and had a thickness of 3 mm. The cervix and bilateral adnexa displayed no abnormalities. The mass was sampled by examining 2 sections /cm. Histological examination of the formalin-fixed, paraffin-embedded sections revealed a marked, predominantly lymphocytic infiltrate involving a well-circumscribed leiomyoma, and a few lymphocytes extended into the adjacent myometrium with an intravascular tumor thrombus. Within the leiomyoma without any mitosis or necrosis, the lymphoid infiltrate was diffuse, and there were only a few recognizable whorled bundles and fascicles of smooth-muscle cells (Fig. 1a). The lymphoid cells consisted of medium- to large-sized cells with abundant, faintly basophilic cytoplasm and round to oval nuclei, which were sometimes convoluted and which contained dispersed nuclear chromatin and one to multiple prominent nucleoli (Fig. 1b). Mitoses were frequent. In the background, infiltration of a few small lymphocytes was observed. Most of the lymphoid cells were positive for CD79α, CD20 (Fig. 2a), PAX5 (Fig. 2b), bcl-2 and MUM-1 but negative for CD3, CD5, CD4, CD8, CD10, bcl-6, CD56, CD30, CD21, CD23, CD138, perforin and Gram β. The proliferative index (based on Ki-67 staining) was high (approximately 50 %) (Fig. 2c). Smooth-muscle cells were positive for desmin and caldesmon (Fig. 2d). To determine the clonality of the lymphocytic infiltrate at the Ig receptor level, immunoglobulin heavy chain gene rearrangement was evaluated. Via polymerase chain reaction (PCR) and subsequent capillary gel electrophoresis, the suspected monoclonal nature of the infiltrate was confirmed.

The tumor was ultimately diagnosed as a stage IE (Ann Arbor) primary DLBCL of the activated B-cell-like (ABC) subtype arising from the uterine leiomyoma (collision tumor).

## Discussion

Although infiltration of the uterus during the course of malignant lymphoma is not rare, few malignant lymphomas originate in the uterus. In a study with a large number of cases, it was reported that malignant lymphomas originating in the uterus accounted for 0.5 % of all extranodal lymphomas and that they were most common in the cervical region. The histological type of these tumors was DLBCL [1, 2]. Even when the uterus is the main clinical lesion site, it is difficult to determine whether the uterus is the primary site or a site of infiltration in an advanced stage [1, 2]. The criteria for assessing the primacy of a DLBCL are designed such that the diagnosis is only correct if the disease is confined to the organ and if there are no signs of DLBCL in other organs at diagnosis or during follow-up [1, 2]. Although a complete CT or PET scan was not available, our case did not progress during a follow-up period of greater than year without receiving any therapy; as a result, this case may be considered as a diagnosis of primary DLBCL.

One peculiarity of this case was the coexistence of DLBCL and leiomyoma. In previous reports, cases of primary T-cell lymphoma arising from a leiomyoma of the uterus was described, and these tumors expressed T-cell antigens (CD3 and CD8) and markers of cytotoxicity (TIA and granzyme) and exhibited monoclonal rearrangement of the T-cell receptor based on molecular analyses [3]. Our case, however, expressed B-cell antigens (CD20, PAX5 and CD79α) but not markers of T-cells or cytotoxicity. Molecular analyses revealed clonal B-cell receptor gene rearrangement (immunoglobulin heavy chain gene rearrangement). Therefore, this is the first case of a proven primary DLBCL arising from a leiomyoma in the uterine corpus.

The most important differential diagnoses in this case included leiomyoma with heavy lymphocytic infiltration and massive lymphoid infiltration simulating lymphoma (lymphoma-like lesion) [4–7]. Regarding the previously reported lesions, the leiomyoma contained a variably dense infiltrate consisting of small lymphocytes, scattered larger lymphoid cells, and, occasionally, numerous plasma cells. Germinal centers were identified in some cases and ranged from large and florid to inconspicuous. Lymphoid infiltration was confined to the leiomyoma or was present to only a minor extent in the adjacent myometrium. Immunohistochemical findings were positive for desmin and leukocyte common antigen and demonstrated a positive reactivity for B-cell and T-cell markers. Additionally, the proportions of B and T cells were nearly equal, and there was no preponderance of either lymphoid cell type [4–7]. In our case, the lymphoid infiltrate extended into the adjacent myometrium and included an intravascular tumor thrombus. Immunohistochemistry showed a preponderance of the B-cell type, and molecular analyses provided evidence for clonal B-cell receptor gene rearrangement. Therefore, we defined the pathological diagnosis as primary DLBCL arising from a leiomyoma. It must be emphasized, however, that florid reactive lymphoid hyperplasia (lymphoma-like lesion) may exhibit clonal IGH gene rearrangement based on PCR [8]. Therefore, it is essential to comprehensively analyze clinical, histologic, immunophenotypic, and genetic features to establish the correct diagnosis.

Because our case had a history of 23 years, it may be hypothesized that the DLBCL evolved as a result of progression from a polyclonal inflammatory population concomitant with the development of a leiomyoma with lymphocytic infiltration or massive lymphoid infiltration, resulting in a collision tumor. However, there is a lack of strong evidence supporting this assumption.

## Conclusions

We report the first documented case of DLBCL concurrent with leiomyoma in the uterus. Although these findings could be purely coincidental, it should be considered that there might be a causal relationship between DLBCL and leiomyoma with lymphocytic infiltration. We could not exclude the possibility that the leiomyoma induced a lymphoid reaction with an extranodal lymphoma, representing the final event of progression of the inflammatory reaction.

### Consent

Written informed consent was obtained from the patients for publication of this Case Report and any accompanying images. A copy of the written consent is available for review by the Editor-in-Chief of this journal.

## Abbreviations

BCR: B-cell receptor
DLBCL: diffuse large B cell lymphoma
PCR: polymerase chain reaction
